# Supplementary figures and images for: Different antigenic distance metrics generate similar predictions of influenza vaccine response breadth despite moderate correlation
Source: PLoS Comput Biol. 2025 Nov 14;21(11):e1013720. doi: 10.1371/journal.pcbi.1013720 (PMC12629490; doi:10.1371/journal.pcbi.1013720)

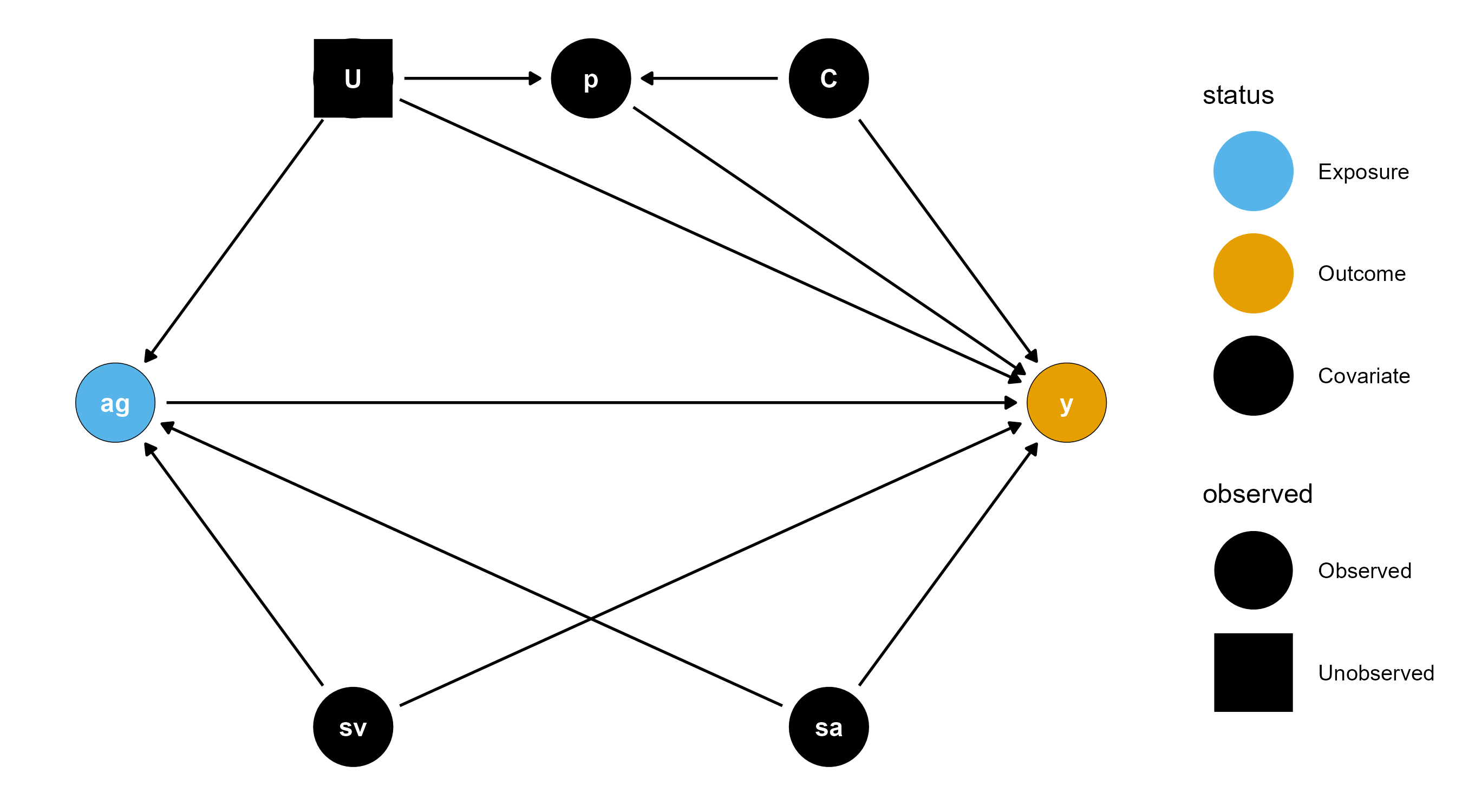

Supplement: S1 Fig — (PNG) [file pcbi.1013720.s013.png]

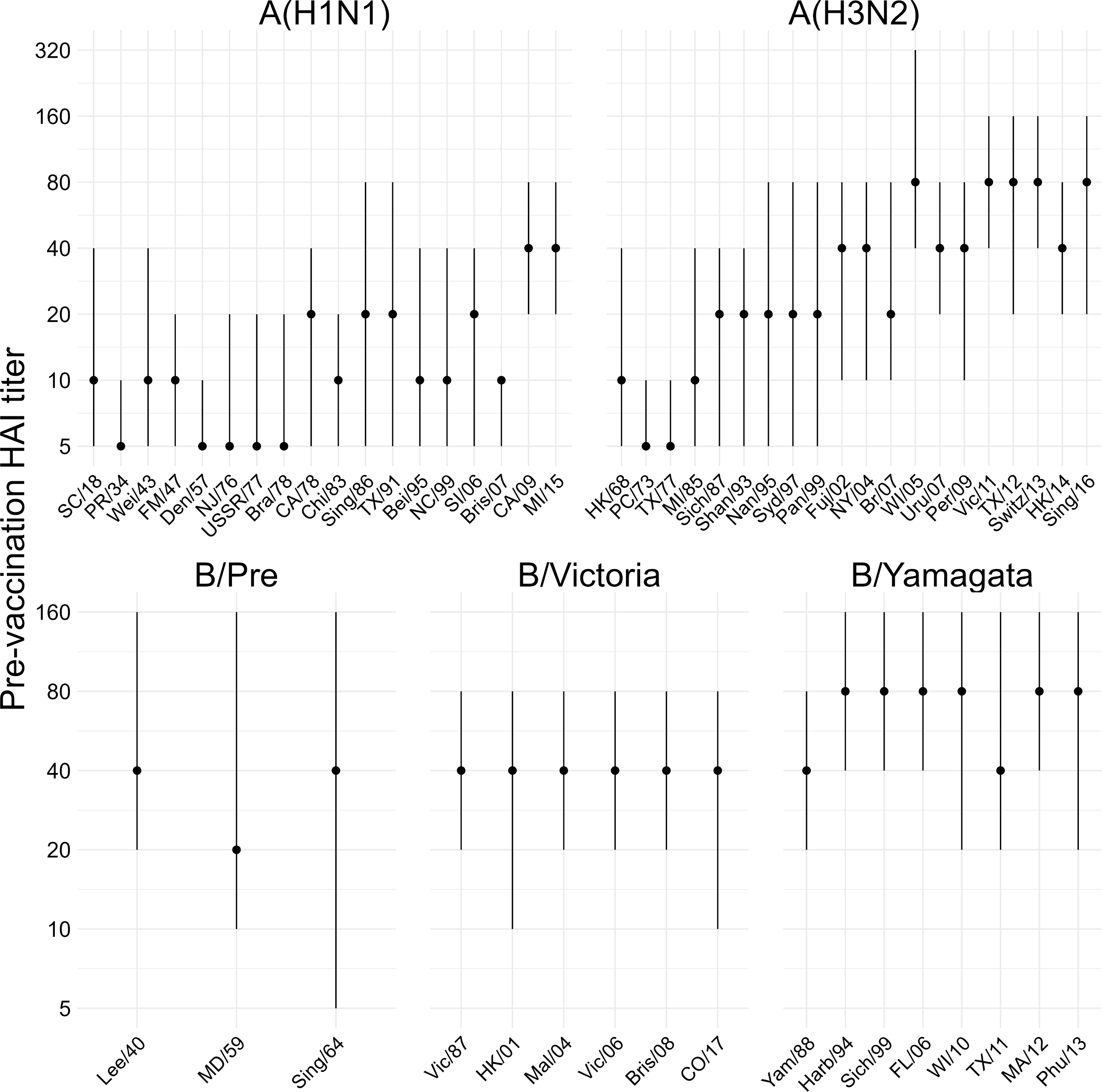

Supplement: S2 Fig — The point shows the median and the line shows the IQR. (TIF) [file pcbi.1013720.s014.tif]

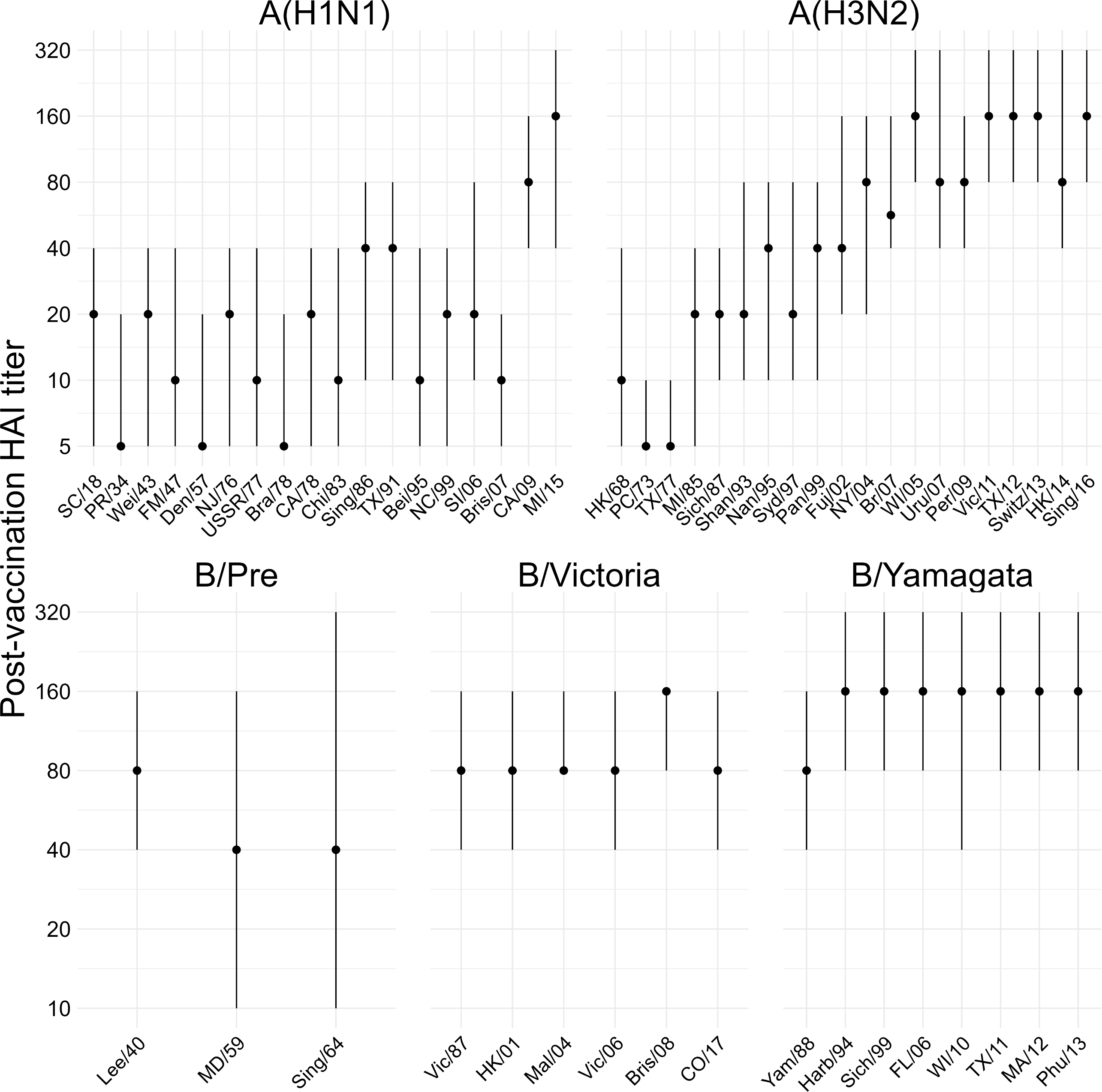

Supplement: S3 Fig — The point shows the median and the line shows the IQR. (TIF) [file pcbi.1013720.s015.tif]

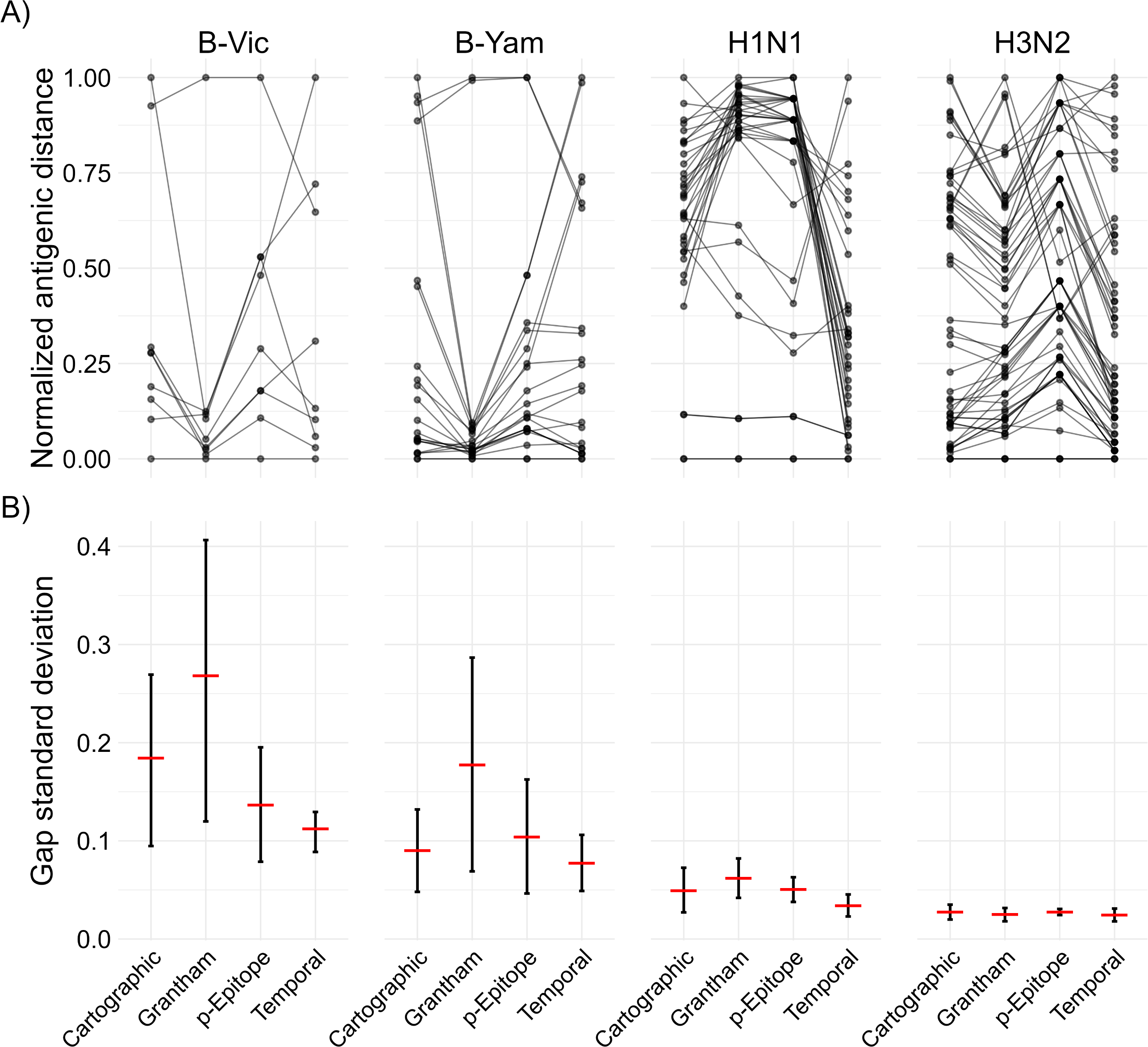

Supplement: S4 Fig — A) Parallel coordinates plot showing how the estimated pairwise antigenic distances change for each of the antigenic distance metrics. Each line in the plot represents one vaccine strain and assay strain pair, and the connected points are the pairwise distance measured under each metric shown on the x-axis. When two lines cross, this indicates that two metrics assigned a different relative order to the pairwise combination. Note that Grantham and especially p-Epitope distances are integer-valued and concentrate measurements to specific points which potentially overlap (temporal distance is also integer valued but has enough spread to avoid a similar issue). B) The gap standard deviation (gap SD) for each subtype and antigenic distance metric. The posterior distribution of gap SDs was calculated using the bayesian bootstrap with reweighting. The red horizontal bar shows the mean of the bootstrap posterior and the error bars show the 95% highest density credible interval (HDCI). (TIF) [file pcbi.1013720.s016.tif]

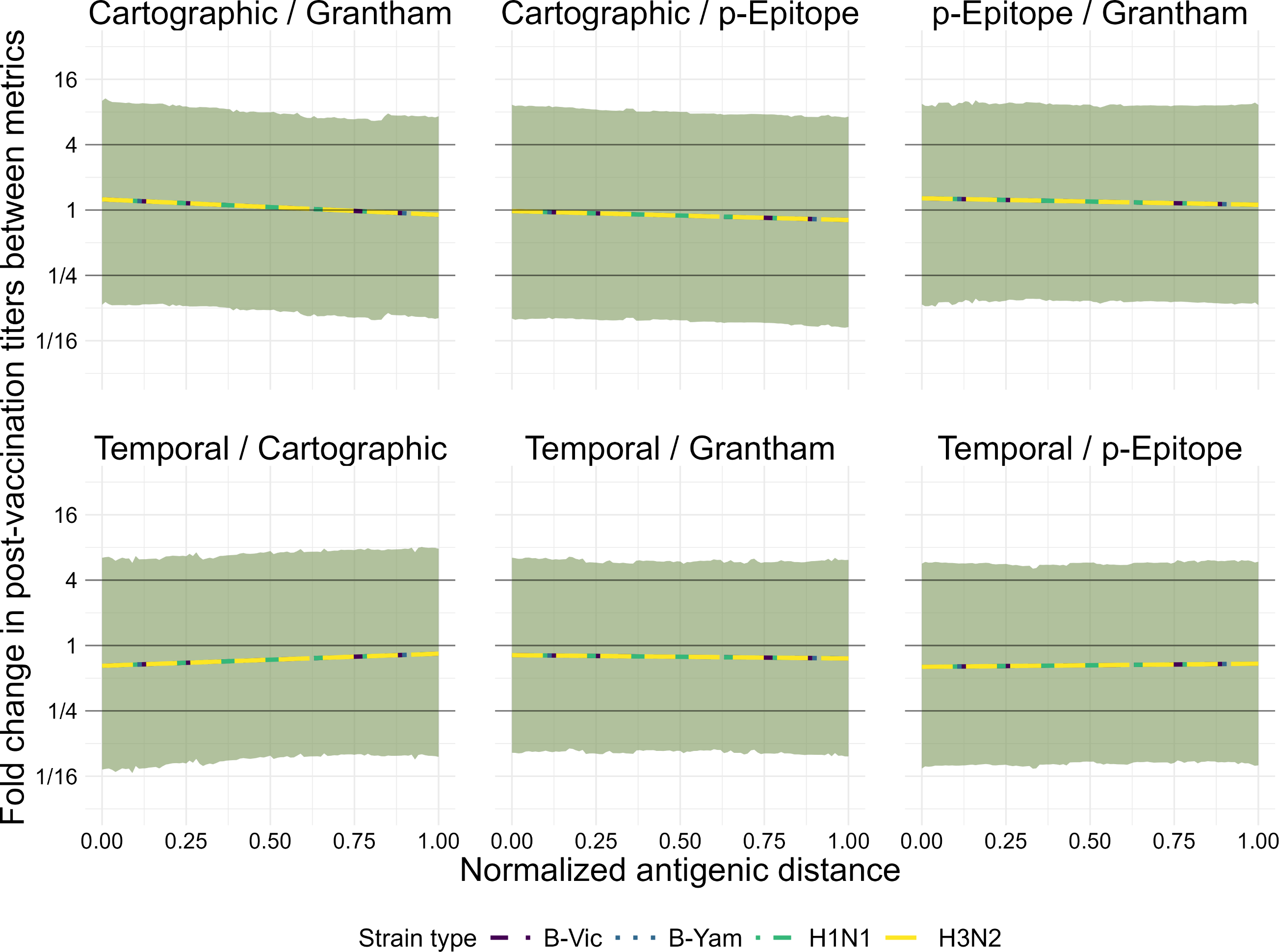

Supplement: S5 Fig — The y-axis shows the fold change in predictive titers between metrics, and the two metrics being compared in each subplot are shown as the subplot labels. Each line represents the predictions for the first metric in the pair at a given antigenic distance value divided by the predictions for the second metric in the pair. Color and linetype correspond to different strain types. The solid black lines on the plot are reference lines at a value of 1 for no effect, and at 4 and 1/4, effect values which would represent a clinically notable deviation in HAI predictions beyond what is expected from measurement error. Lines represent the mean of the posterior distribution of the contrast and the colored ribbons represent the 95% highest density credible interval (HDCI) for each strain type in each subplot. (TIF) [file pcbi.1013720.s017.tif]

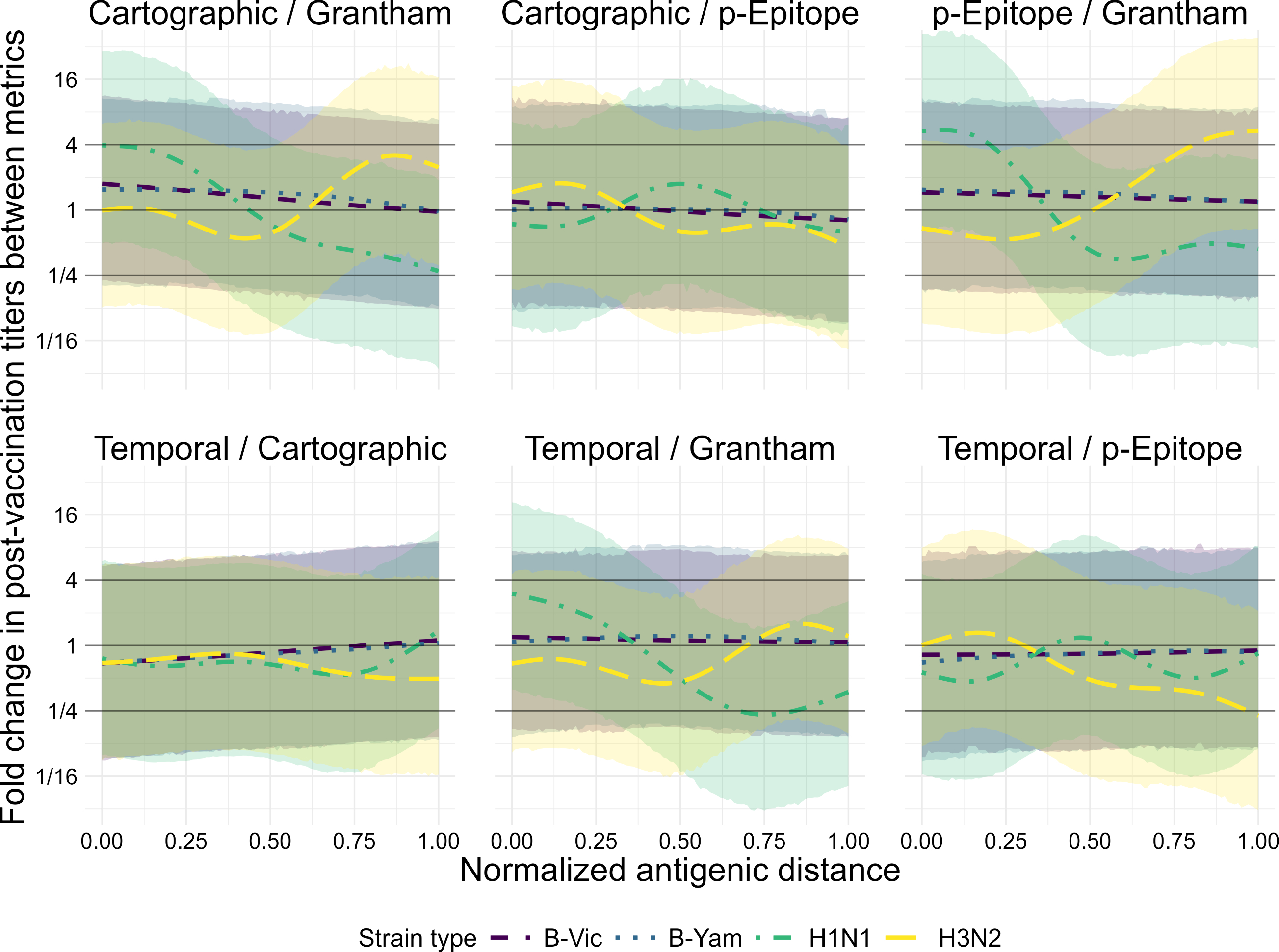

Supplement: S6 Fig — The y-axis shows the fold change in predictive titers between metrics, and the two metrics being compared in each subplot are shown as the subplot labels. Each line represents the predictions for the first metric in the pair at a given antigenic distance value divided by the predictions for the second metric in the pair. Color and linetype correspond to different strain types. The solid black lines on the plot are reference lines at a value of 1 for no effect, and at 4 and 1/4, effect values which would represent a clinically notable deviation in HAI predictions beyond what is expected from measurement error. Lines represent the mean of the posterior distribution of the contrast and the colored ribbons represent the 95% highest density credible interval (HDCI) for each strain type in each subplot. (TIF) [file pcbi.1013720.s018.tif]

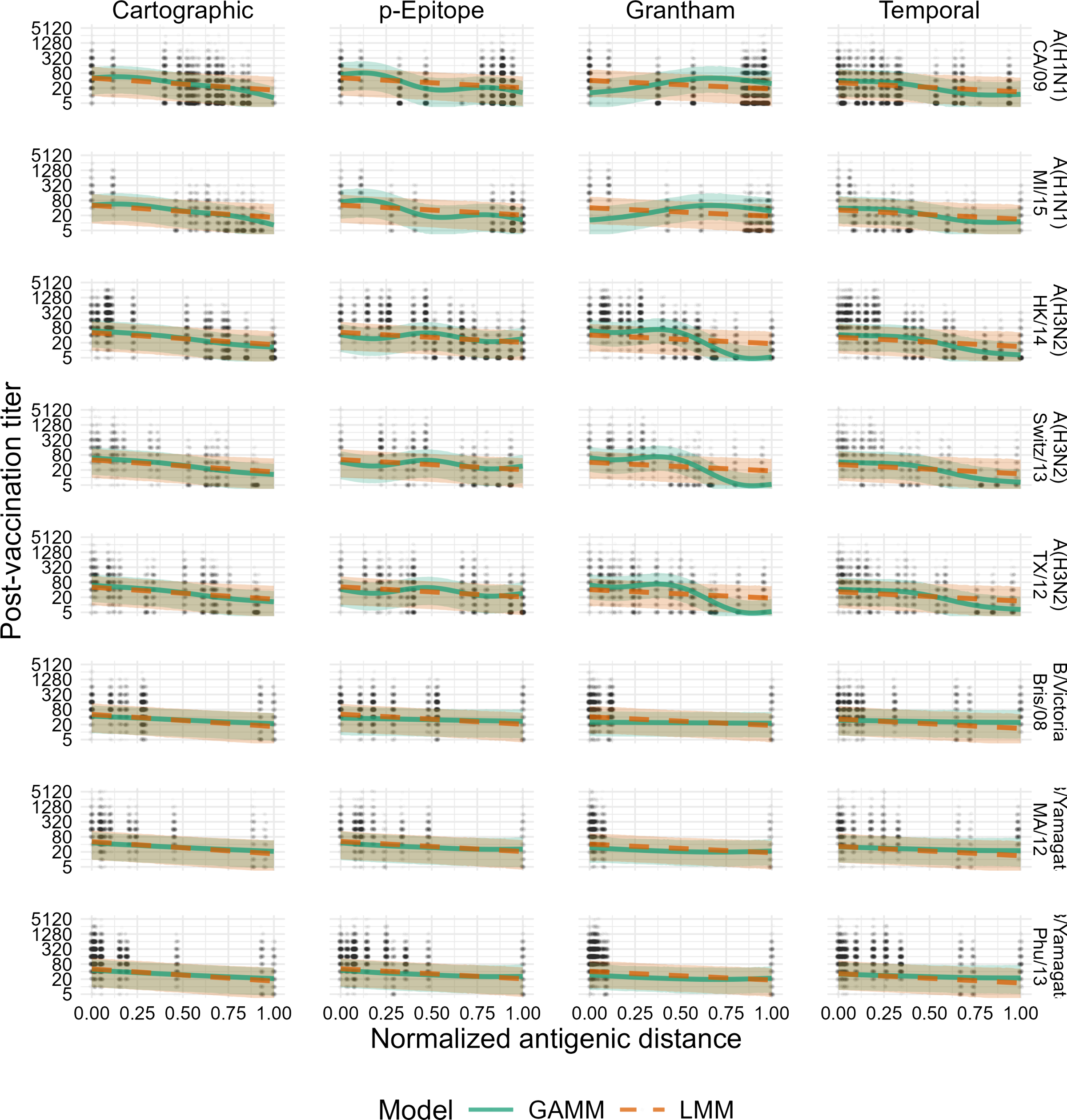

Supplement: S7 Fig — Solid green lines and green ribbons show the mean and 95% highest density continuous interval (HDCI) for GAMM predictions. Dashed orange lines and orange ribbons show the mean and 95% HDCI for LMM predictions. Circular points show the data values. Each subplot shows the model predictions for a particular subtype (changes by row) and distance metric (changes by column). Outcomes shown on the plot are predicted post-vaccination titers for an average individual to an average strain. (TIF) [file pcbi.1013720.s019.tif]
