# Supplementary material for: Different antigenic distance metrics generate similar predictions of influenza vaccine response breadth despite moderate correlation
Source: PLoS Comput Biol. 2025 Nov 14;21(11):e1013720. doi: 10.1371/journal.pcbi.1013720 (PMC12629490; doi:10.1371/journal.pcbi.1013720)
Supplement: Attachment — Submitted filename: plos-response-to-reviewers.docx [file pcbi.1013720.s021.docx]

**Response to reviewers for “Different antigenic distance metrics generate similar predictions of influenza vaccine response breadth despite moderate correlation”**

Reviewer 1

This manuscript describes comparison of several different measures of antigenic distance between influenza vaccine and assay strains and assesses their ability to predict vaccine response. The authors find that even though the different measures are only slightly correlated, they have similar predictive power when it comes to vaccine efficacy. I enjoyed reading this manuscript and particularly appreciate the very thorough descriptions of the methodology found in the supplement. I have only a few very minor comments:

1. The authors often use "which" when they should use "that". Please check correct use of these.

2. Line 235: "difference" should be "different"

3. Line 246: "outcome" is misspelled.

We thank the reviewer for their kind comments, and we greatly appreciate the reviewer’s examination of our supplementary material. We have corrected the grammatical and typographical errors raised by the reviewer above.

4. Line 343: AIC and BIC are not defined in the manuscript.

We have revised this sentence to not include any acronyms.

5. Line 370: What do the authors mean by "casually adjusted"?

We thank the reviewer for noting this humorous typo. We meant “causally adjusted” referring to the graphical causal framework we used for developing our models, but we elected to rephrase this sentence instead of just fixing the mistake.

6. Tables 4 and 5. Is is possible to use a smaller font in the metric column so that these names are not split on two lines?

We agree with the reviewer that this change would be useful. We have adjusted the formatting in our draft tables, but we ultimately defer all table formatting to the journal as there is no guarantee our tables will remain in the same layout in the published version of the article.

We thank the reviewer once more for their time and their complementary review.

Reviewer 2

The paper considers an interesting and important question — the evaluation the choice of influenza strains when updating flu vaccines. The antigenic distance between 2 strains is typically measured by antigenic cartography. In this approach groups of ferrets are immunized with each of these 2 strains and the extent to which the serum binds the homologous (immunizing) strain vs the heterologous (other) strain gives us a measure of the antigenic distance between these strains. This is a involved and rather difficult approach and the paper considers the use of other measures for the antigenic distance between two (vaccine) strains and how this affects the breadth of vaccine-induced antibody responses the vaccine elicits. They use 3 metrics for antigenic distance between 2 strains: Grantham, p-Epitope and Temporal. 
 
They show many interesting results including the following. 
1. Table 2 and Figure 1. There is low ICC across the different antigenic distances for responses to H1 and B lineages and better correlation only for the responses to H3(N2). They might want to discuss why?

We briefly discussed this point in paragraph two of the discussion, and we have substantially expanded this commentary given the reviewer’s interest. The discussion of low correlation. Notably, we feel that this question is predominantly immunological in nature, so our answers are only speculative. Since we have relatively few heterologous strains for influenza B, we focus our speculation on the difference between H3N2 and H1N1 influenza A.

H3N2 only represents one “major” genetic cluster (used loosely, not in a technical sense) which has evolved over time in a ladder-like pattern, as opposed to the two clusters of H1N1 which have emerged over the past century – the 2009-like pandemic strain, which is similar to the 1918 pandemic strain, and the pre-pandemic seasonal lineage. One major synapomorphy of the 2009-like lineage relative to the prior lineage is an insertion mutation, which is unlikely to significantly increase the p-Epitope and Grantham distances, but could radically change the cartography map. Additionally, the 2009-like H1N1 viruses are more similar to the 1918 viruses than they are to, say, the strains that emerged in the 1980s, which distorts the temporal distance. H3N2 does not have this feature because, generally speaking, novel H3N2 strains are more distant to each previous dominant strain (so far). So perhaps the issue is that there is no suitable metric that currently exists for accurately quantifying the genetic distance between H1N1 strains when indels are present (a problem that could also extend to the influenza B strains).

2. Figure 2. They then consider how the “breadth of the response” (measured by the post-vaccination titer to the homologous vs heterologous strains) depends on antigenic distance for the different strains and the different measures of antigenic distance. 
3. Figure 3. They show that using different antigenic metrics yields similar results for the breadth of the response. 
 
This suggests that the simpler measures for antigenic distance might provide comparable results to antigenic cartography. This is an important and potentially useful result.

We thank the reviewer for their kind comments, and we agree that the final result noted by the reviewer is the main point we wanted to emphasize in our manuscript.

I have a couple of minor questions: 
1. Can some combination of the different measures of antigenic distance provide improved prediction?

The reviewer raises an interesting question that we have spent a lot of time considering. In principle, yes, some combination of metrics could provide improved prediction. However, we believe that developing a model for combining metrics is outside the scope of our paper, and prediction was not our main goal. We believe that building such a model, while valuable, constitutes a separate research product. Most importantly, it would be important to have data on a strong clinically relevant outcome, such as probability of infection, for which making predictions would be worthwhile. In our case, the comparison of antibody responses for different metrics does not provide a clear gold-standard outcome.

However, we have now revised our discussion to mention this point as a future goal with the potential to advance the field.

2. How does prior infection history affects the results. This is clearly a question for subsequent studies but might be mentioned in the discussion.

We mention this as a limitation of our study in the discussion (paragraph beginning at line 458), but the reviewer is correct that we do not fully discuss the importance of prior infection history. Of course prior infection history is a complicated topic and one of the main topics of interest in the field of influenza vaccination, and only now are studies being designed that can capture individual-level effects of infection history on vaccination immunogenicity.

Statistically, controlling for individual exposure (infection and vaccination) history could allow us to eliminate nuisance variation in our study, providing better estimates of the effect of antigenic distance; if we knew more about exposure history, we might be able to reduce the amount of between-subject variability. Exposure history does not causally confound the relationship we analyze (the effect of antigenic distance on immunogenicity) because an individual’s exposure history cannot affect the antigenic distance between the current vaccine strain and the strains used for HAI assays. While exposure history at the population level partially shapes the space of possible strains that could evolve, the assay strain used is a treatment that is not dependent on any feature of individual exposure history.

We have added several sentences to the discussion to briefly summarize these points, but we include our detailed reasoning here for the reviewer.

Overall I enjoyed reading the paper and believe it will be a nice addition to the literature.

Again, we thank the reviewer for their insightful questions and positive remarks.
